# A Luciferase Immunosorbent Assay Based on Attachment Glycoprotein for the Rapid and Easy Detection of Nipah Virus IgG Antibodies

**DOI:** 10.3390/microorganisms12050983

**Published:** 2024-05-14

**Authors:** Xinyue Li, Yuting Fang, Xinyi Huang, Yongkun Zhao, Chengsong Wan

**Affiliations:** 1 BSL-3 Laboratory, Guangdong Provincial Key Laboratory of Tropical Disease Research, School of Public Health, Southern Medical University, Guangzhou 510515, China; lxy22120085@smu.edu.cn (X.L.); axy988@smu.edu.cn (Y.F.); h22320235@smu.edu.cn (X.H.); 2 Key Laboratory of Jilin Province for Zoonosis Prevention and Control, Changchun Veterinary Research Institute, Chinese Academy of Agricultural Sciences, Changchun 130122, China

**Keywords:** Nipah virus, attachment glycoprotein, luciferase immunosorbent assay, IgG, detection

## Abstract

Nipah virus (NiV) is a virulent zoonotic disease whose natural host is the fruit bat (*Pteropus medius*), which can coexist with and transmit the virus. Due to its high pathogenicity, wide host range, and pandemic potential, establishing a sensitive, specific, and rapid diagnostic method for NiV is key to preventing and controlling its spread and any outbreaks. Here, we established a luciferase immunosorbent assay (LISA) based on the NiV attachment glycoprotein (G) to detect NiV-specific immunoglobulin G by expressing a fusion protein of nanoluciferase (NanoLuc) and the target antigen. Sensitivity analysis was performed and compared to an indirect enzyme-linked immunosorbent assay (ELISA), and specificity and cross-reactivity assessments were performed using NiV-positive horse serum and Ebola virus-, Crimean–Congo hemorrhagic fever virus-, and West Nile virus-positive horse sera. The optimal structural domain for NiV detection was located within amino acids 176–602 of the NiV G protein head domain. Moreover, the LISA showed at least fourfold more sensitivity than the indirect ELISA, and the cross-reactivity results suggested that the LISA had good specificity and was capable of detecting NiV-specific immunoglobulin G in both mouse and horse serum. In conclusion, the establishment of a rapid, simple NiV LISA using the G protein head domain provides a resource for NiV monitoring.

## 1. Introduction

Nipah virus (NiV), an emerging pathogen of bats, is a single-stranded RNA virus with negative polarity and belongs to a family of the genus *Henipavirus* along with Hendra virus (HeV) [1]. It was first reported and named during an acute encephalitis outbreak in Malaysia in 1998, and infections with NiV can cause severe neurological and respiratory disorders [2]. Since 2001, successive NiV outbreaks have been reported in neighboring countries, including Singapore, Bangladesh, and India [3]. Recently, a novel outbreak of NiV was reported in the Kerala state of India in August 2023, with a high mortality rate of 40–75% and 30 reported cases of infection [4].

The NiV genome is approximately 18.2 kb in size and consists of six genes that encode six structural proteins: nucleocapsid (N), phosphoprotein (P), matrix protein (M), fusion protein (F), glycoprotein (G), and RNA polymerase (L). The *P* gene also encodes three non-structural proteins through RNA editing or optional open reading frames [5]. Phylogenetic analysis based on the complete *N* and *G* genes has shown that NiV can be divided into strains of at least two different genotypes [6], NiV–Malaysia and NiV–Bangladesh. Furthermore, the two evolutionary clades have different routes of transmission; an intermediate host caused the NiV outbreak in Malaysia, whereas the outbreaks in Bangladesh have been associated with human-to-human transmission [7,8]. The head and lungs are the two major target organs for NiV infection. Infected patients may develop fever, headache, muscle pain, vomiting, and sore throat [9], followed by dizziness, drowsiness, altered consciousness, and neurological symptoms that indicate acute encephalitis [10]. The high lethality and zoonotic nature of NiV pose a significant threat to public health security, along with its pandemic potential [11].

Laboratory testing is crucial for the diagnosis of infection, as no typical clinical signs of disease are easily noted during NiV infection. Virus isolation is considered the gold standard for detection, but its application is limited to the requirement for a biosafety level 4 (BSL-4) laboratory [12]. Molecular diagnosis is the preferred method for confirming acute NiV infection because of its high sensitivity and specificity. Unfortunately, it should be noted that the viral RNA load gradually decreases in the body over the course of infection, and it has not yet been determined whether NiV spreads during the incubation period [13,14]. Serological tests have the advantages of being cost-effective and rapid and are suitable for large-scale screening and epidemiological studies.

The virulence effect of NiV begins with membrane fusion of the virus with host cells. This fusion primarily relies on the viral G and F proteins, important glycoproteins that facilitate virus binding to the ephrinB2 and ephrinB3 receptors on the host cell surface. This binding leads to the fusion of the viral and cellular membranes, promoting the viral invasion of cells [15,16,17]. The NiV G protein consists of an N-terminal transmembrane domain, a neck domain, and a C-terminal receptor-binding head domain [18]. Additionally, it has highly specific and immunogenic antigenicity that can still exist even when only the head domain is retained [18], which is why the NiV G protein serves as the primary immunodominant target for neutralizing antibodies against NiV [19]. Therefore, the NiV G protein is frequently utilized in the development of vaccines and serologic assays against NiV [20]. Indirect enzyme-linked immunosorbent assays (ELISAs) have been developed to detect NiV and HeV [21]. These ELISAs show reproducibility in detecting anti-NiV immunoglobulin G (IgG). However, the application of ELISA for monitoring NiV across a wide range of hosts may be limited because of the requirement for changing the specific secondary antibody depending on the species. Although a solid-phase blocking ELISA can detect only neutralizing antibodies against NiV and can be applied to different species, its preparation requires virus-infected cellular antigens, which can be limited due to experimental conditions [22].

In this study, a new antigen–antibody-binding method, luciferase immunosorbent assay (LISA), was established to detect NiV infection. This novel technique utilizes nanoluciferase (NanoLuc, Promega, Madison, WI, USA), which generates a highly intense and bright luminescent signal and is 150 times more active than the firefly or Renilla luciferases [23], thus amplifying the antigen–antibody-binding signal and improving the sensitivity of the assay. Moreover, there is no need for antibody preparation and purification, as the G protein is used as the antigen for the detection of simulated clinical serum samples. This approach can quickly and sensitively recognize NiV-specific IgG antibodies and shorten the detection period. Therefore, in this study, the sensitivity and specificity of the LISA were evaluated to determine the capability of the method to track the zoonotic transmission of NiV and its diagnostic potential.

## 2. Materials and Methods

### 2.1. Samples and Reagents

Serum samples from mice immunized with NiV G-protein (*n* = 9) and negative mice (*n* = 9) were prepared in our laboratory. Sera from NiV-, Ebola virus (EBOV)-, Rift Valley fever virus (RVFV)-, Crimean–Congo hemorrhagic fever virus (CCHFV)-, and West Nile virus (WNV)-positive horses were provided by Changchun Veterinary Research Institute, Chinese Academy of Agricultural Sciences. Sera from patients infected with Chikungunya virus (CHIKV) and Dengue virus (DENV) were provided by Guangdong Center for Disease Control and Prevention (China), and normal human and hepatitis C virus (HCV) sera were provided by Nan Fang Hospital, Southern Medical University, Guangdong, China. These sera were kept in our laboratory and were validated in previous studies. A bicinchoninic acid (BCA) protein assay kit was purchased from Beyotime (Shanghai, China). The pNLF1-N (Promega, Madison, WI, USA, N1351) plasmid was maintained in our laboratory for subsequent vector construction. A Nano-Glo luciferase kit (Promega, N1120, USA) was purchased from Promega. HEK 239T cells were maintained in our laboratory and cultured in 5% CO_2_ at 37 °C using Dulbecco’s Modified Eagle Medium (DMEM; Gibco, Waltham, MA, USA) containing 10% fetal bovine serum.

### 2.2. Design and Construction of Recombinant Plasmids Based on G Proteins

Recombinant plasmids were generated using the mammalian NanoLuc luciferase expression vector pNLF1-N. The gene sequence of the NiV G protein (strain Malaysia prototype) was inserted into pNLF1-N by seamless cloning with a total length of 1806 nt (GenBank accession number AF212302.2). To further study its structure and antibody-binding sites, we designed five amino acid sequences, including a full-length G protein sequence, the G protein head domain (aa 176–602) and three recombinant protein sequences, with a 6×His tag added to the C-terminus to verify that the fusion proteins were properly expressed. These five amino acid sequences were designed to explore the optimal antibody-binding site for the G antigen. We used the primers listed in Table 1 for amplification and confirmed the ligation and sequence of each recombinant plasmid by means of double enzyme digestion and sequencing.

### 2.3. Expression of the Luciferase Fusion Antigen

Cryopreserved HEK 293T cells were thawed rapidly by immersing the cryopreservation tubes directly in a static water bath set at a constant temperature of 37 °C after removal from liquid nitrogen. The thawed cells were cultured in a 37 °C incubator using DMEM supplemented with 10% FBS in a cell culture flask. HEK 293T cells were inoculated in six-well plates, and the experiment was started after the cell density reached 70–90%. Transfection was performed using Lipofectamine 3000 transfection reagent (Invitrogen, Waltham, MA, USA) according to the reagent instructions, with 2.5 µg of recombinant plasmid DNA per well. Cell culture supernatants were discarded after 48 h of transfection, and the cells were washed twice with phosphate-buffered saline (PBS) to remove the cell culture medium. The whole-cell extracts were prepared using radioimmunoprecipitation assay buffer (Beyotime, Shanghai, China) with phenylmethanesulfonyl fluoride (Beyotime, Shanghai, China) and protease inhibitor cocktail (Solarbio, Beijing, China). And the lysates were then centrifuged at 15,000 rpm for 30 min to collect the supernatants, which were stored at −80 °C.

### 2.4. Western Blot

The protein concentration of the lysate was determined using the BCA kit to calculate the loading volume of the target proteins and the negative control. Equivalent amounts of protein from each sample were separated using sodium dodecyl sulfate–polyacrylamide gel electrophoresis (SDS–PAGE) in precast 8–12% gradient Future PAGE^TM^ gels (ACE bio, Nanjing, China) and transferred to polyvinylidene difluoride (PVDF) membranes (Millipore, Burlington, MA, USA). Then, the membranes were blocked with 5% non-fat dry milk and incubated with rabbit anti-His (D3I1O, diluted 1:1000) (CST, San Antonio, TX, USA), and mouse anti-β-actin (diluted 1:10,000) (Proteintech, Rosemont, CA, USA) antibodies overnight at 4 °C, followed by incubation with horseradish peroxidase (HRP)-conjugated anti-rabbit or anti-mouse secondary antibodies (Bioss, Beijing, China) at room temperature. Finally, protein bands were visualized using hypersensitive chemiluminescence (ECL) reagent (Bioworld, Nanjing, China) and detected using a Tanon imaging system.

### 2.5. Serum Preparation

The NiV G protein used for immunization was expressed and purified by ReadCrystal Co., Ltd. (Suzhou, China). The full-length extracellular domain of NiV-G was cloned into the pI-sec baculovirus expression vector, and the protein was expressed using SF9 cells [24]. The purified protein was used as an immunogen and mixed with Freund’s complete adjuvant at a ratio of 1:1 to prepare simulated positive serum samples. A total of nine 4- to 6-week-old female BALB/c mice were chosen for subcutaneous immunization with 30 µg of protein. Booster immunizations were administered on days 14 and 28 following the initial immunization. Additionally, nine 4- to 6-week-old female BALB/c mice were included as a negative control group and subcutaneously immunized with 400 µL PBS. Blood samples were collected on days 0 (before immunization), 28, and 42. After the samples were coagulated for 1 h at 25 °C, sera were collected by means of centrifugation (5000 rpm, 30 min, 4 °C) and stored at −80 °C to be used for the subsequent establishment of the LISA. 

### 2.6. Establishment of the NiV-G-LISA

Protein G (Genscript, Nanjing, China) was used at 5 mg/mL and diluted to 5 µg/mL in PBS (0.01 M, pH 7.4), and 100 µL per well was added to white microtiter plates and stored at 4 °C overnight. After the plates were washed three times using PBS containing 0.05% tween (PBST), 5% skim milk powder (Sangon Biotech, Shanghai, China) was prepared and added as a blocking solution to each well for 1 h of incubation at 37 °C. PBST was used to wash the plates three times after the blocking step, and excess liquid was carefully removed. Then, the samples to be tested were diluted with 2% skimmed milk at 1:100, and 100 µL of each diluted sample was added to the wells of the plate, which was then incubated at 37 °C for 1 h. Following incubation, the plate was washed five times with PBST, and excess liquid was removed. Next, the cell lysates containing the luciferase fusion antigen were diluted with 2% skimmed milk powder (at a 1000-fold dilution of the antigen), 50 µL of the diluted antigen was added to each well, and the plate was incubated at 37 °C for 30 min. At the end of the incubation, the plates were washed three times and patted as dry as possible after the final wash. Finally, 50 µL of luciferase substrate was added to each well according to the reagent instructions, and the relative fluorescence intensity (RFI) was measured within 2 h using a fluorescence photometer. The RFI of the negative control was calculated, and the positivity cut-off was determined to be twice the mean value of the negative control. All samples were measured in at least three replicate wells.

To avoid differences in the transfection efficiency and protein expression of different batches of preparations, we measured the luciferase activity of crude cell lysates to determine the RFI, which was usually between 10^4^ and 10^6^. The NiV antigen was always added in each reaction with 10^5^ RFI for the LISA. In addition, we included positive and negative controls in each reaction plate to keep the results consistent and reproducible.

### 2.7. ELISA

A previously reported indirect ELISA [21] was used for sensitivity comparison with the LISA. The NiV G protein used for coating antigen was the extracellular domain (aa 71-602) expressed through SF9 cells. The main experimental steps were as follows: NiV G protein was diluted using 0.01 M PBS (pH 7.4) and added at 100 ng/well (100 µL volume) to plates overnight. The plates were then blocked with 5% skim milk in 0.01 M PBS for 2 h at 37 °C and washed three times with PBST. Mouse serum samples were added, and goat anti-mouse IgG conjugated to HRP (Bioss, Beijing, China) was added at a dilution of 1:50,000. The optical density (OD) reading at 450 nm was taken using a multifunctional microplate instrument (Tecan, Shanghai, China). The positivity cut-off value was set at OD = 0.34, which was two times the mean value of the negative control.

### 2.8. Statistical Analysis

All data were processed using GraphPad Prism software version 9.0. Data (San Diego, CA, USA) are presented as the mean ± standard deviation (SD). An unpaired *t* test was used to determine statistical significance. *p* < 0.05 was considered to indicate statistical significance.

## 3. Results

### 3.1. Expression of Different Fragments of the NiV G Protein Luciferase Fusion Antigen

**Figure 1:**
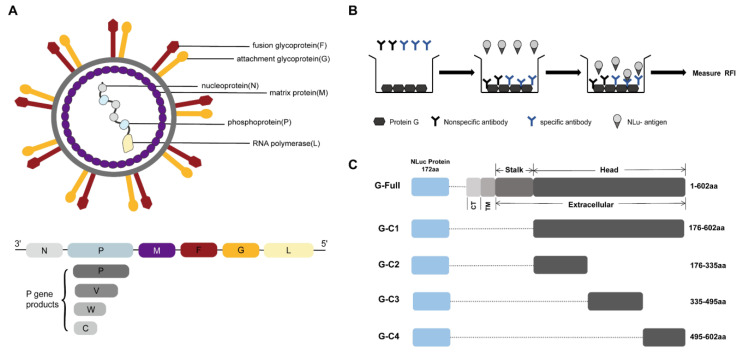
(**A**) Schematic diagram of the Nipah virus structure. (**B**) Schematic protocol for the luciferase immunosorbent assay (LISA). A protein G-coated microtiter plate is used to bind the recombinant protein to a specific antibody, and then a luciferase substrate is added to produce a fluorescent signal. (**C**) Schematic design of the antigen detection fragments. The G protein sequence was fused to the end of the NanoLuc sequence and then cloned into pNLF1-N to construct the recombinant plasmid. CT: cytoplasmic tail region, TM: transmembrane region.

NiV is spherical in shape, with its G and F proteins, which promote membrane fusion, embedded in the viral monolayer envelope and M proteins arranged under the envelope. Highly pathogenic NiV consists of six genes, which continuously encode six proteins (Figure 1A).

**Figure 2:**
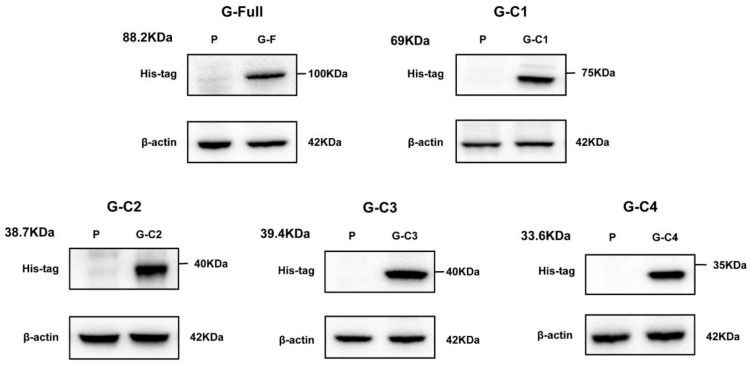
Recombinant plasmids were validated by means of Western blotting. Rabbit anti-His antibodies were used to detect the five fusion proteins, G-Full, G-C1, G-C2, G-C3, and G-C4. Beta-actin was used as an internal control. P, empty plasmid pNLF1-N.

We acquired five testing fragments to establish the NiV-LISA based on the structure of the G protein. The five recombinant plasmids were as follows: G-Full (full-length G), G-C1 (containing the head domain of the G protein), and the remaining three, G-C2, G-C3, and G-C4 (containing truncated fragments of the head domain), which were confirmed by means of double restriction enzyme digestion and agarose gel electrophoresis (Figure 1B,C). Then, the indicated plasmids were transfected into HEK 293T cells to express the luciferase fusion proteins, with further validation confirmed by means of Western blot. These results confirmed the successful construction of these recombinant plasmids expressing their indicated proteins (Figure 2). These recombinant plasmids based on the G antigen were then evaluated for use in the NiV-LISA for IgG detection.

### 3.2. Establishment of the LISA for the Detection of NiV IgG Antibody

Because of the high infectivity and pathogenicity of NiV, virus isolation must be conducted in BSL4 conditions. Moreover, it is challenging to obtain positive sera from patients and infected animals. Therefore, we utilized antigen-immunized mice to acquire positive serum samples. These samples served as essential material support for the subsequent development of the NiV-LISA technology.

**Figure 3:**
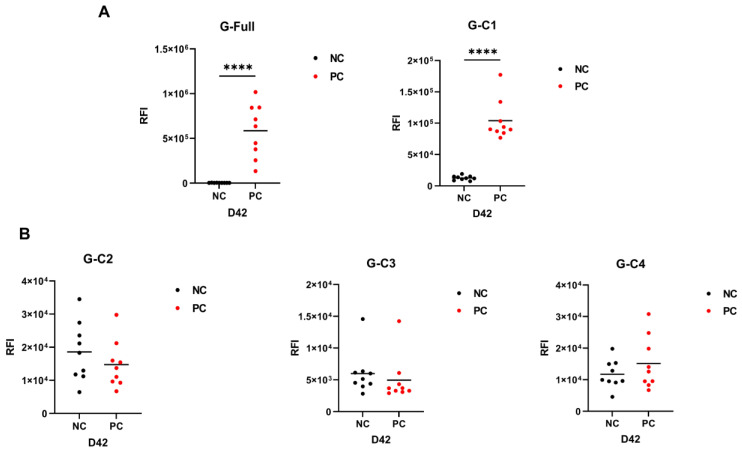
Nine NiV G-protein immunized mouse serum samples and nine negative control mouse serum samples were detected using LISA. (**A**) Schematic diagram of G-Full and G-C1 fragments with detection efficacy. (**B**) Schematic diagram of the detection of three fragments designed based on NiV G protein head structural domain. The serum dilution ratio was 1:100, and ****, *p* < 0.0001, unpaired *t* test. NC: negative control; PC: positive control.

The mean value of the RFI of the negative control was first determined, and the positivity cut-off value was defined as twice the RFI of the negative control. Any sample with an RFI above the positivity cut-off value was considered positive for NiV infection. Then, we performed LISA to detect the NiV IgG antibody based on all five recombinant test fragments. Serum samples obtained 42 days post immunization were analyzed. The full-length fragments, G-Full and G-C1, were able to completely detect nine positive serum samples with 100% detection rates, and the positive detection RFI was significantly higher than the negative control (*p* < 0.0001, Figure 3A). These results suggested that the efficacy of the G-Full and G-C1 recombinant fragments for the detection of mouse NiV IgG appeared to be optimal.

### 3.3. Optimal Structural Domains for NiV IgG Antibody Detection

**Figure 4:**
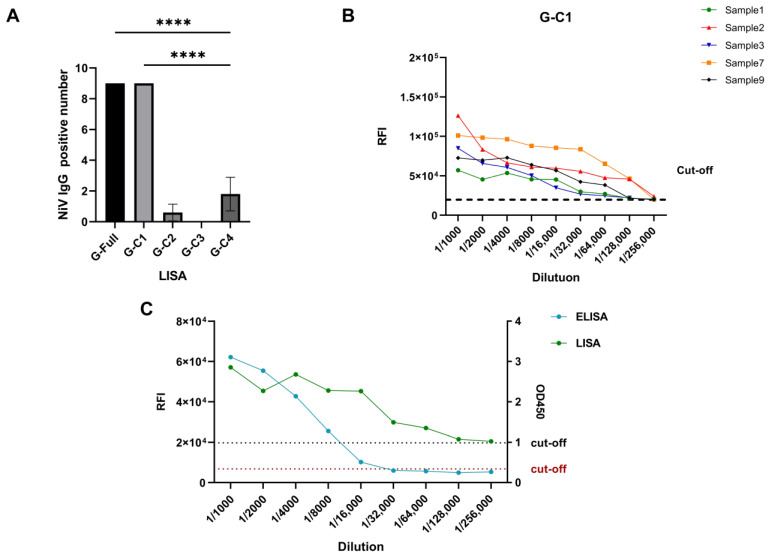
Evaluation of the detection efficacy of the G-C1 fragment. (**A**) Comparison of the numbers of detected fragments from the five tested fragments of the NiV G antigen. ****, *p* <   0.0001, one-way ANOVA test. (**B**) Sensitivity analysis of G-C1. Five positive sera were randomly selected and diluted to different folds, with the positivity cut-off value of twice the mean value of the negative control. (**C**) A sensitivity analysis was conducted comparing the LISA with an indirect ELISA. Measurements were carried out using a gradient dilution of serum, and the positivity cut-off value for positive results is indicated by the dashed line.

To further explore the optimal detection domain of the antigen, we divided the G-C1 fragment into three fragments to characterize the binding domain of the antibody produced against the NiV G protein. Though the G-C1 fragment demonstrated better detection capabilities, fragments G-C2, G-C3, and G-C4 were unable to effectively differentiate between positive and negative samples (Figure 3B). The numbers of positive samples detected using LISA were counted (Table 2): G-C2 detected one positive sample, G-C3 failed to detect any positive samples, and G-C4 detected two positive samples; in contrast, G-Full and G-C1 could detect all positive samples (*p* < 0.0001, Figure 4A).

Notably, the complete expression of the full-length G protein exhibited superior efficacy in detecting the NiV IgG antibody. This efficacy may be attributed to the sufficient exposure of crucial antibody-binding sites following the intact translation of the G protein or the comprehensive characterization of receptor-binding sites within the full-length structure. Moreover, the head domain G-C1 (176–602 aa) also showed the ability to distinguish between positive and negative samples, which may indicate that there are a wide range of NiV IgG antibody-binding sites in this domain that could trigger obvious immune responses, which are proper targets for antigen–antibody binding.

### 3.4. Sensitivity and Cross-Reactivity Analysis of the G-C1-LISA

We next focused on the ability of G-C1 to detect NiV-positive serum-specific IgG. To further valuate the efficacy of G-C1, we performed gradient dilutions of three positive sera with a maximum dilution of 1:256,000, with the lowest concentration for detection of 0.0235 ng/mL. The G-C1 sequence failed to detect IgG antibodies in all positive samples at dilutions of 1:128,000–1:256,000, indicating that the lower limit of detection fell within this range (Figure 4B). Notably, a gradient dilution of Sample 1, measured using an indirect ELISA, failed to detect a positive sample at a dilution of 1:32,000. This result indicates that the LISA was approximately four to eight times more sensitive than the ELISA (Figure 4C).

**Figure 5:**
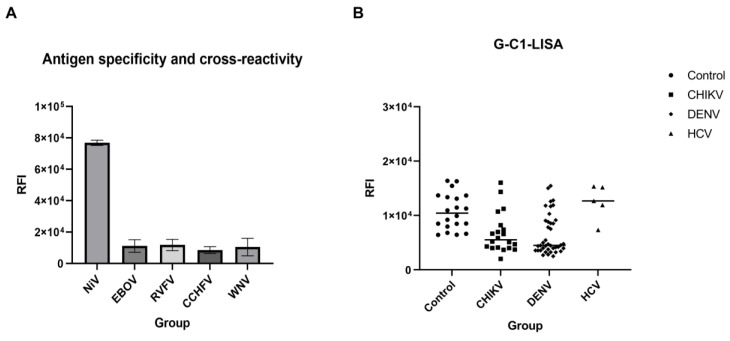
Cross-reactivity of the G-C1-LISA. (**A**) Validation was performed using positive horse sera from NiV, Ebola virus (EBOV), Rift Valley fever virus (RVFV), Crimean–Congo hemorrhagic fever (CCHFV), and West Nile Virus (WVN) infections. (**B**) Serum samples from healthy humans were used as the controls, and the G-C1-LISA was evaluated for cross-reactivity using positive sera from patients infected with CHIKV, DENV, and HCV.

Additionally, we also investigated the cross-reactivity of the G-C1-LISA using validated positive sera from horses infected with NiV, EBOV, RVFV, CCHFV, and WNV. The RFI of NiV-positive horse sera was significantly higher than that of the positivity cut-off value. In addition, no other virus-positive (that is, EBOV-, RVFV-, WNV-, and CCHFV-positive) horse sera were scored as positive, suggesting that the LISA-based G-C1 can specifically react with NiV-positive sera and is capable of cross-species testing for both mouse and horse serum (Figure 5A). Moreover, 20 CHIKV-positive samples, 40 DENV-positive samples, and 5 HCV-positive samples from infected patients were tested according to the established G-C1-LISA, and 20 sera from healthy individuals were tested as negative controls, which showed that the G-C1 fragments did not detect specific IgG in CHIKV, DENV, and HCV as compared to the negative controls (Figure 5B), indicating that the G-C1 fragment corresponding to the head structure had good specificity.

## 4. Discussion

NiV is a highly pathogenic virus carried by bats that can be transmitted to humans either directly or indirectly via an intermediate host, and has shown evidence of persisting and causing widespread human-to-human transmission in South and Southeast Asia [25]. As with other paramyxoviruses, the NiV G protein plays an important role in infection. The G and N proteins are crucial immunogenic proteins of NiV, and both react with sera from patients recovering from viral infections. It is worth noting that only the NiV G protein exhibits reactivity with sera from patients experiencing late-onset recurrent NiV infections accompanied by complications, despite the advantages of using the N protein for early serological diagnosis, as it stimulates the body to produce high-titer antibodies early in infection [26,27]. Additionally, the NiV G protein serves as an envelope protein of NiV and is the main target protein for inducing the host to generate a protective adaptive immune response [28]. Thus, we developed a rapid and simple LISA based on the NiV G protein, which could help to realize the rapid serological diagnosis of NiV.

The head structural domain (G-C1) of NiV demonstrated significant detection efficacy, consistent with the results reported by Wang [18]. This may suggest that the G head domain contains important antigenic epitopes. In our study, we performed three replicate experiments for the G-Full and G-C1 fragments. However, the values of RFI in the G-Full fragment had a discrete distribution in a single experiment though they significantly exceeded the positivity cut-off value. Additionally, the structure characteristics of the G-Full fragment probably determine the exposure of primary antigen–antibody-binding sites during the process of translation and folding. Considering the same efficacy to detect the NiV IgG antibody, we focused on the G-C1 fragment. The G-C1 fragment was able to detect all positive samples and exhibited a lower limit of detection in the dilution range of 1:128,000–1:256,000, and the sensitivity of the LISA was at least four times higher than that of an indirect ELISA, confirming that the structural domain of the head of the NiV G protein is the optimal structural domain for the detection of NiV IgG. These results indicate that maintaining the integrity of the antigenic epitopes in this structural domain and presenting a correctly translated and folded protein spatial structure in this region can improve the detection efficacy.

To further explore the key areas of G-C1 for detection, we divided the head domain into three antigen-detecting fragments, G-C2, G-C3, and G-C4, but none of these fragments showed the same detection efficacy as G-C1. This failure was possibly due to the fact that we truncated the head region, disrupting the antibody-binding epitopes and leading to decreased detection. Taken together, these findings further support that the head structural domain of the NiV G protein is able to fully utilize its antigenicity when it is structurally intact, allowing the antibody-binding site to be fully characterized. In addition, due to the presence of hydrophobic regions that cannot be fully expressed in a prokaryotic system, our LISA utilizes an eukaryotic expression vector for detection, which likely better expresses the G antigens, exposing more antibody-binding sites and improving the detection efficacy, thus realizing the ultra-sensitive diagnostic capability of the LISA [29,30].

As the natural host of NiV, fruit bats can co-evolve with the virus and facilitate cross-species transmission by means of virus spill-over, infecting both humans and animals [31,32]. Therefore, cross-species serological testing is advantageous for monitoring and preventing the spread of zoonotic diseases. However, the traditional ELISA relies on species-specific enzyme-linked secondary antibodies, limiting the range of their application. In addition, it would be challenging to obtain specific antibodies from certain hosts, including mites and bats. This point also applies to NiV, which has a wide range of hosts. In contrast, the LISA does not require species-specific labeled secondary antibodies for detection [33]. To conduct cross-species testing and validate the cross-reactivity, we utilized NiV-positive horse sera and serum samples from other viral infections exhibiting clinical symptoms similar to those of NiV infection [34]. Our results indicated that the G-C1 fragment is capable of discrimination between NiV mouse IgG and horse or human IgG against other viruses (EBOV, RVFV, CCHFV, WNV, CHICKV, DENV, and HCV). However, as NiV-positive samples are not easily accessible but critical for specificity evaluation, more sera from multiple species (especially human) will be required to fully validate and optimize the LISA.

In conclusion, we developed a novel serologic LISA based on the NiV G protein with high sensitivity and rapidity, which could be applicable for on-site and late-stage NiV detection. In addition, this proof of principle assay could theoretically be used for large-scale screening in natural foci to assess the potential epidemic risk of NiV.

## Figures and Tables

**Table 1:** Recombinant antigen primer DNA sequences.

Amplified Gene	Primer Name	Primer Sequence
G-C1 G-C1	F R	TCGCTTCCGAATTCAGAGCTCAAGAAGGGGTGAGCAATCTAGTAGGATTAC AATTGGGCCCAAATCTAGATTAATGGTGATGGTGATGGTGTGTACATTGCTCTGG
G-C2 G-C2	F R	TCGCTTCCGAATTCAGAGCTCAAGAAGGGGTGAGCAATCTAGTAGGATTAC AATTGGGCCCAAATCTAGATTAATGGTGATGGTGATGGTGAAGTTGATGTTGATTG
G-C3 G-C3	F R	TCGCTTCCGAATTCAGAGCTCAACTTGCCCTACGAAGTATCGAGAA ATTGGGCCCAAATCTAGATTAATGGTGATGGTGATGGTGTCTAGGGCATTGTGATT
G-C4 G-C4	F R	ATCGCTTCCGAATTCAGAGCTCAAAGATTCAATACATGTCCAGAGATCTG AATTGGGCCCAAATCTAGATTAATGGTGATGGTGATGGTGTGTACATTGCTCTGG

**Table 2:** Comparison of the number of NiV G antigen fragments detected.

	G-Full	G-C1	G-C2	G-C3	G-C4
Anti-NiV IgG positive *	1/2/3/4/5/6/7/8/9	1/2/3/4/5/6/7/8/9	4	/	1/3/4
Anti-NiV IgG negative	/	/	1/2/3/5/6/7/8/9	1/2/3/4/5/6/7/8/9	2/5/6/7/8/9

* Anti-NiV IgG positive (Sera from NiV G-protein-immunized mice). /: No samples were detected negative (G-Full, G-C1); no samples were detected positive (G-C3).

## Data Availability

Data are contained within the article.
